# Pollutants, microbiota and immune system: frenemies within the gut

**DOI:** 10.3389/fpubh.2024.1285186

**Published:** 2024-05-10

**Authors:** Pierluigi Rio, Antonio Gasbarrini, Giovanni Gambassi, Rossella Cianci

**Affiliations:** Department of Translational Medicine and Surgery, Catholic University of Sacred Heart, Fondazione Policlinico Universitario A. Gemelli, IRCCS, Rome, Italy

**Keywords:** microbiota, dysbiosis, immune system, pollution, colorectal cancer, inflammatory bowel diseases

## Abstract

Pollution is a critical concern of modern society for its heterogeneous effects on human health, despite a widespread lack of awareness. Environmental pollutants promote several pathologies through different molecular mechanisms. Pollutants can affect the immune system and related pathways, perturbing its regulation and triggering pro-inflammatory responses. The exposure to several pollutants also leads to alterations in gut microbiota with a decreasing abundance of beneficial microbes, such as short-chain fatty acid-producing bacteria, and an overgrowth of pro-inflammatory species. The subsequent intestinal barrier dysfunction, together with oxidative stress and increased inflammatory responses, plays a role in the pathogenesis of gastrointestinal inflammatory diseases. Moreover, pollutants encourage the inflammation-dysplasia-carcinoma sequence through various mechanisms, such as oxidative stress, dysregulation of cellular signalling pathways, cell cycle impairment and genomic instability. In this narrative review, we will describe the interplay between pollutants, gut microbiota, and the immune system, focusing on their relationship with inflammatory bowel diseases and colorectal cancer. Understanding the biological mechanisms underlying the health-to-disease transition may allow the design of public health policies aimed at reducing the burden of disease related to pollutants.

## 1 Introduction

Human health is closely related to environment and it is well-known how some environmental factors can be responsible for different diseases.

Pollution is constituted by harmful materials, called pollutants, spread into the environment. These materials include, among others, pesticides, petroleum hydrocarbons, human and veterinary medicaments, cosmetics, heavy metals, pathogenic bacteria, per-and poly-fluoroalkyl substances (PFAS), and microplastics (MPs) (1).

Pollutants can be considered a leading, often neglected, global health risk and the strongest environmental determinant of illness with about 9 million related early deaths worldwide (16% of all deaths), and more than 260 million disability-adjusted life-years, in 2015 (2, 3). For this reason, national income influences the impact of pollution on non-communicable disease (NCD) mortality. While, in high-income countries, the leading determinants of NCD mortality are mainly lifestyle-associated, since pollution has been brought in part under control, in low-income countries, NCD mortality depends mainly on pollution (4). NCDs associated to environmental pollution have affected and continue to affect many individuals worldwide, with an estimated mortality rate ranging from 20 to 25% (5). Data collected in the southwestern Iran, a region characterized by dust storms and high levels of air pollution, correlate the exposure to particulate matter (PM) and the incidence of cardiovascular diseases (6). In the same geographical area, a reduced prevalence of chronic obstructive pulmonary disease (COPD) was observed during the period 2009–2013, following a decreasing trend of PM environmental levels (7). These results confirm the etiological role of air pollutants, especially PM, in COPD (8). Moreover, a close association between air pollution exposure and hospital admissions for both cardiovascular and respiratory diseases has been demonstrated (9, 10).

Among various biological mechanisms involved in pollution-dependent human diseases, a crucial role is played by the immune activation in response to toxicants. For instance, after a sustained exposure to particulate matter below 2.5 μm in diameter (PM_2.5_), inflammation and oxidative stress have been identified as some of the main drivers of the cardiovascular, respiratory and systemic consequences. In fact, the uptake of PM_2.5_ by the lung immune cells is responsible for the release of inflammatory and oxidative mediators, able to induce damage even in distant regions of the body (11). Moreover, heavy metals, particularly lead, have proved to promote the release of pro-inflammatory cytokines, dysregulate inflammatory enzymes (e.g., cyclooxygenases), and increase the production of reactive oxygen species (ROS) that disrupt some cell constituents, such as phospholipids through lipid peroxidation (12). Furthermore, the resulting inflammatory status impairs the cell-mediated and humoral immune responses, thus making the exposed individual susceptible to autoimmunity, sensitization and disease (5, 12). For instance, environmental pollution can trigger immunotoxicity through the phosphoinositide pathway, which is involved in the onset of autoimmune disorders (5).

In recent decades, microbiome research has significantly advanced due to novel insights into the roles of microflorae in health and disease. The gut microbiota (GM) is a complex ecosystem encompassing bacteria, archaea, fungi and parasitic organisms that can be found in the human gastrointestinal tract (13). The GM composition is extremely variable among individuals resulting from different factors, such as age, sex, antibiotic administration, diet and behavioral habits (14, 15). Similarly, environmental pollutants have demonstrated an impact on the GM composition, leading to dysbiosis and altering the intestinal homeostasis (13). At gut level, where a complex interplay between gut microbiota and immune system takes place, an imbalance between these two factors may cause altered gut permeability, inflammation and ultimately intestinal diseases (16).

Overall, from human activities derive several forms of pollution (17) with heterogeneous effects on human health. In this narrative review, we will discuss on the impact of pollutants on immunity and gut microbiota composition; then, we will consider their potential relation with inflammatory bowel diseases (IBD) and colorectal cancer (CRC), focusing on specific pollutants.

## 2 Pollutants and immune system: roots of chronic inflammation

Pollution is one of the primary concerns of modern society for its various effects on human health, despite a widespread lack of awareness. In Europe, public perceptions of air pollution are subjective, frequently deviating from the scientific community’s definitions, and common people often underestimate its potential harm (18).

Overall, industrialization, urbanization, population growth, deforestation, and motor vehicle production have contributed to increase the environmental exposure to air pollutants. This exposure has proved to impair human health at different levels by causing, for instance, asthma, chronic pulmonary disease, dizziness, headache, cardiovascular dysfunction, immune dysregulation, gastrointestinal disorders and cancer (19). Borsi et al. (20) evaluated the consequences on health of the exposure to air pollutants, such as PM, nitrogen dioxide (NO_2_), sulfide dioxide (SO_2_) and ozone (O_3_), in a highly urbanized area in Iran. The authors found a significant association between increased environmental exposure to air pollutants, cardiovascular mortality, and hospitalization for cardiovascular or respiratory disease (20). Moradi et al. (21) came to similar results by investigating the health effects related to PM in the air of Ardabil. Moreover, Faraji Ghasemi et al. (22) detected a significant ecological and health risk level due to atmospheric PM-bound heavy metals in the Persian Gulf. A recent analysis of data from the Global Burden of Diseases Study 2019 revealed that the disease burden related to air pollution decreased in North Africa and the Middle East from 1999 to 2019, in spite of reliable geographical differences. However, the authors detected a growing exposure to PM in these countries, where reducing pollution to the minimum risk exposure levels would have increased life expectancy (23).

The immune system, which is involved in the fight against pathogens, embraces a considerable number of cells and pathways, in order to orchestrate immune responses. Pollutants can affect the activity of specialized immune cells, such as phagocytes, dendritic cells (DCs) and neutrophils, that play a role in both arms of immune response (24).

The inhalation of air pollutants can perturb the immune regulation in lungs, by upregulating the antigen presentation on DCs with subsequent production of inflammatory cytokines, such as interleukin (IL)-6, able to upregulate T cell response and inhibit T regulatory cells (Tregs) (25). Moreover, air pollutants can weaken Tregs activity through epigenetic modifications, such as hypermethylation of the Forkhead box transcription factor 3 (Foxp3) (26). Air pollution also dysregulates antiviral immunity and increases adaptive immune responses through T helper type 2 (Th2) and Th17 cells. These immune processes take part to the worsening of COPD and asthma after a relevant air pollution exposure (27–29).

Furthermore, antigen oxidation may contribute to the loss of self-tolerance in many autoimmune diseases (30, 31). A population-based cohort study by Ma et al. (32) defined the exposure to small gaseous pollutants, particularly carbon monoxide (CO), methane (CH_4_) and nitric oxide (NO) as an independent risk factor for primary Sjögren’s syndrome. Among the underlying molecular mechanisms, the IL-6 signalling pathway seems to be involved (32). Additionally, an Italian retrospective observational study found that a prolonged exposure to particulate matter below 10 μm in diameter (PM_10_) increases the risk of rheumatoid arthritis, whereas the exposure to PM_2.5_ increases the risk of rheumatoid arthritis, connective tissue diseases and IBD (33).

Pollution can dysregulate responses against microbes by promoting the viral adhesion to respiratory mucosa, through the expression of CD54 by epithelial cells (34–36) or by reducing phagocytic function of macrophages (37, 38). In a large study conducted on healthcare workers during the COVID-19 pandemic in Milan (Italy), the exposure to NO_2_ has been associated with an increased risk of SARS-CoV-2 infection, but also with the development of higher antibody levels in positive individuals. These data confirm a causative role of air pollutants in viral infections and their capacity to influence the immune responses against pathogens (39).

Air pollutants can exert their toxic effect also in the central nervous system (CNS), after reaching it through the bloodstream. At this level, they can directly damage neurons or dysregulate the normal functions of microglia and brain immune cells (40). Microglia are the resident macrophages of the CNS, which also play an key role in initiating adaptive immune responses (41, 42). In CNS, toxic air pollutants can stimulate the microglia to fight them causing, consequently, brain inflammation. For this reason, air pollutants have been considered an environmental factor in the onset and relapses of inflammatory diseases involving the brain, such as multiple sclerosis (40, 43).

Similarly, water and land pollutants, such as both primary and secondary microplastics, can trigger inflammation (44). As showed by animal studies, the intake of these materials is responsible for gut inflammation and microbiota composition disruption, leading to systemic diseases (45, 46). Oxidative stress is the crucial factor in the inflammatory pathways activated by particulate matter and microplastics. Reactive oxygen species while at low doses act as signalling molecules and can be used by the host immune system against pathogens (e.g., phagocytosis), at high concentrations, induce oxidative damage, tissue dysfunction and inflammation (47). Harusato et al. (48) came to unexpected results, investigating the intestinal impact in mouse models of polyethylene terephthalate-derived particulate microplastics (PM-PET). After a prolonged exposure to minimal doses of PM-PET, neither the gut barrier nor the number of lymphocytes, neutrophils and macrophages were altered. However, a downregulation of immune regulatory genes, together with an effect on gut immune cell metabolisms occurred (48). After ingestion, the uptake of MPs by gut cells generally occurs through endocytosis. Hence, MPs cause intracellular oxidative stress, impair mitochondrial function and induce apoptosis. Consequently, the coated-MPs released by the lysed cells activate pathogen recognition receptors (PRRs), such as Toll-like receptors (TLRs), and trigger inflammation. Moreover, the MPs endocytosis by neutrophils causes cell death and the release of neutrophil extracellular traps (NETs) that retain MPs in body (49). Additionally, MPs easily interact with proteins (e.g., immunoglobulins, complement and coagulation proteins) that form a protein-corona, and with chemicals and pathogens that increase the MP toxicity (49). Wolff et al. (50) have recently evaluated the effects of MPs, such as polystyrene (PS), poly methyl methacrylate (PMMA) and amino-modified PS (PS-NH_2_), on human immune cells *in vitro*. Macrophages and phagocytic dendritic cells have proved to be highly sensitive to MPs, whereas T-cells are less affected by cytotoxicity and have a higher expression of markers of activation (50).

At the gut level, pollutants exert their harmful effects through the production by immune cells of several cytokines (e.g., Transforming Growth Factor beta (TGF-β) and IL-17), and changes in innate immunity receptors, such as TLRs. For example, among the pollutants harmful to the gut, petrol and pesticides directly promote the release of Tumour Necrosis Factor (TNF) and IL-17. Moreover, pesticides exert their immunotoxicity through ROS production, leukocyte apoptosis or cell cycle impairment (51). Particulate matter also induces loss of intestinal barrier function, through the increased expression of pro-inflammatory cytokines, with simultaneous increasing of epithelial permeability, and the disruption of gut microbiota and short-chain fatty acid (SCFA) homeostasis (52).

Furthermore, polycyclic aromatic hydrocarbons (PAHs) impact on the epithelial Th17/Tregs equilibrium and the aryl hydrocarbon receptor (AhR) (24). AhR is a xenobiotic receptor, able to react to various pollutants and promote detoxification. Its chronic activation generates immunotoxicity with consequences on Tregs vs. Th17 cell differentiation (53, 54). Zhang et al. (55), dosing urinary levels of hydroxylated PAHs (OH-PAHs) found substantial correlations with respiratory damage.

Among the different inflammatory pathways activated in response to environmental pollutants, such as benzo(a)pyrene (BaP) – typically found in tobacco smoke – and nanoplastics, it is worth considering micro-RNAs (miRNAs), whose altered expression may lead to significant gene expression changes (56–58).

The exposure to heavy metals, that can be naturally found in the environment (e.g., air, water, soil, etc.) as organic or inorganic compounds, but also as products of industrial and combustion processes, has important consequences on immune system and immune regulation. For example, cadmium, a biologically non-essential heavy metal, has toxic effects on immune cells, such as the promotion of pro-inflammatory cytokines, the inhibition of phagocytic activity, the apoptosis of neutrophils and lymphocytes, the reduced activity of natural killers (NK) cells, a possible genotoxicity, ROS production and oxidative stress (59). According to Akash et al. (60), heavy metals reduce lipid and amino acid metabolism through oxidative stress.

Bisphenols (BPs), employed in plastics industry, act as endocrine disruptors. The bisphenol A (BPA) exposure enhances macrophage expression of growth hormone secretagogue receptor (GHSR) and the release of inflammatory mediators, such as IL-6 and C-C motif chemokine ligand 2 (CCL2), in colonic mucosa. GHSR ligand is ghrelin, a nutrient-sensing hormone, able to regulate metabolism and inflammation (61).

Bosch et al. (62), administered diesel exhaust particles (DEP) to mice, achieving a prevalence of inflammatory phagocyte subpopulations and a reduction of C-C chemokine receptor type 2 (CCR2) anti-inflammatory/resident macrophages in the intestine. Moreover, the inflammasome nucleotide oligomerization domain (NOD), leucine-rich repeat (LRR) and pyrin domain-containing protein 3 (NLRP3) was activated (62).

In the last decades, several studies investigated the impact of pollution on carcinogenic risk. In a comprehensive review of literature, Yousefi et al. (63) reported that the exposure to air pollutants significantly increases lifetime cancer risk in both adults and children. Moreover, Khalaf et al. (64) showed that heavy metals (e.g., arsenic, mercury, vanadium and lead) could accumulate at urinary level and be responsible for the development of urological abnormalities, such as kidney, ureter, bladder or prostate tumors. This happens due to the excretory role of the urinary system, which is constantly in contact with toxicants and chemicals, in order to remove them from the blood (64).

A large body of evidence associates the air pollution to the incidence of infertility in both men and women, lower birth rates correlating with the exposure level, as well as different cancers, such as gastrointestinal, respiratory, gynaecologic and brain cancers (65, 66).

Guo et al. (67) in a large cohort study have recently explored the relationship between lung cancer and the prolonged exposure to PM and NO_2_, identifying the existence of a causal link.

Pollution-dependent increased risk of cancer is based on similar mechanisms described for systemic inflammation. One of these mechanisms is the altered DNA methylation in response to some environmental pollutants (68, 69). Another risk factor is oxidative stress with a consequent excessive activation of immune system against pathogens and the loss of self-tolerance due to an inhibition of Tregs. These conditions are responsible for low-grade chronic inflammation which can promote the onset of neoplastic diseases (24).

Yang et al. (70) demonstrated that the exposure to PM_2.5_ promotes the proliferation and migration of human lung adenocarcinoma cell line A549. In these cells, after PM_2.5_ exposure, the authors described an upregulation of Wnt/β-catenin signalling pathway and a greater expression of a miRNA, called miR-582-3p (70). Indeed, several studies showed a PM-induced rearrangement of metabolic and inflammatory pathways (e.g., nuclear factor kappa B (NF-kB), oxidative stress, altered miRNA expression), responsible for cancer onset and progression (71). Moreover, Wang et al. (72) found that PM_2.5_ exposure activate the AhR in lung cancer cells and, subsequently, the transmembrane protease serine 2 (TMPRSS2)-IL18 pathway, which is involved in tumour proliferation and metastasis.

Buñay et al. (73) demonstrated that polychlorinated biphenyls (PCBs) upregulate ACAT1 (microsomal enzyme acetyl-coenzyme A-acetyl transferase 1) through the activation of AhR, thus increasing prostate cancer aggressiveness.

A study evaluating the pulmonary effects of a massive environmental oil spill supports the hypothesis of the onset of inflammation, cell death and potential genotoxicity due to oil or dispersants exposure (74).

PFAS are fluorinated aliphatic chemicals that have been extensively studied also as carcinogens, since they can lead to hormonal disorders and cause epigenetic perturbations (75). Several studies have identified multiple sources of PFAS exposure for firefighters, such as aqueous film-forming foams, air and dust at the fire scene (76). Importantly, firefighters have showed a higher cancer risk compared to the general population due to the occupational exposure not only to PFAS, but also to other chemical agents, such as benzene and benzo[a]pyrene. Among the cancer types, gastrointestinal and urological tumors have been described. The main carcinogenic mechanisms seem to be the altered DNA methylation that can inactivate some tumour-suppressor genes, changes in miRNA expression, and metabolic imbalance (77) (Table 1).

**Table 1:** Pollutants, immune dysregulation and possible impact on human health.

Pollutants	Pathogenic mechanisms	Health consequences	References
*Particulate matter*	Pro-inflammatory cytokines production Upregulated presentation of antigens Impaired phagocytosis Inhibition of Tregs Epigenetic changes in Tregs, Th2 and Th17 Oxidative and nitrosative stress Gut microbiota dysbiosis Increased gut permeability	Asthma COPD exacerbations Gut inflammation Cancer	(24, 52, 78)
*Microplastics*	Oxidative stress Transcriptional and metabolic changes in immune cells Gut microbiota dysbiosis Potential genotoxicity	Gut inflammation Cancer	(45, 48, 79–81)
*Pesticides*	Pro-inflammatory cytokines production Cell death Alterations in gene expression Potential genotoxicity	Inflammation Cancer	(74, 82, 83)
*Bisphenols*	Endocrine disruptor Increased macrophage GHSR and inflammatory mediators in colonic mucosa Gut microbiota dysbiosis Intestinal barrier disruption Carcinogenesis Induction of EMT	Gut inflammation Cancer and altered response to cytotoxic therapy	(61, 84, 85)
*Heavy metals*	Immune dysregulation Oxidative stress Changes in microbiome and metabolome Enzyme inhibition Cell cycle impairment DNA damage	Type 2 diabetes Antibiotic resistance Gut inflammation Cancer	(59, 86–88)

Tregs, regulatory T cells; Th2, type 2 T helper lymphocyte; Th17, type 17 T helper lymphocyte; COPD, chronic obstructive pulmonary disease; GHSR, growth hormone secretagogue receptor; EMT, epithelial to mesenchymal transitions; DNA, deoxyribonucleic acid.

## 3 Pollution and gut microbiota

The gut microbiome with its millions encoding genes is considerably larger than the human genome (89). The intestinal bacterial species mostly belong to six phyla: Firmicutes, Bacteroidetes, Actinobacteria, Proteobacteria, Fusobacteria, and Verrucomicrobia (90). The gut microbiota is dynamic and presents substantial interindividual and intraindividual variations, particularly in response to foods, medicaments, and also pollutants (91). It is interesting to note the intricate xenobiotic–microbiota–host interactions; on one hand, bacteria are influenced by xenobiotics, but, at the same time, they are involved in their biochemical alterations. Moreover, by modifying the chemical structures of pollutants, intestinal microbes may modulate their harmful effects on host (92, 93).

As described by Jin et al. (94) in mice, the prolonged exposure to polystyrene MP can impair gut barrier through a reduction of mucus production. Moreover, it can modify the composition of gut microbiota, reducing microbial diversity and the amount of Actinobacteria, and can affect metabolic pathways, such as amino and bile acid metabolisms (94). Additionally, MPs release in the ecosystem their toxic chemical additives (e.g., *hexabromocyclododecane, dibutyl phthalate, and copper ions*) and act as vectors for microbes that form biofilms on their surface, thus causing further GM imbalance (95). Tamargo et al. (96) exposed human GM to concentrations of MPs closer to the estimated daily dietary intake, showing that specific gut microbial species (e.g., *Pseudomonas aeruginosa, Escherichia coli, and Staphylococcus epidermidis*) adhere and form biofilms on them. According to the authors, in this way intestinal bacteria could defend themselves, establish relationships and act as a community (96).

Persistent organic pollutants (POPs), such as tetrachlorodibenzofuran, tetrachlorodibenzo-p-dioxin and polychlorinated biphenyls, can reduce bacterial metabolic activity, influencing, among others, carbon, pyruvate, lipid and protein metabolisms (97). Some POPs are chemical additives added to plastics during their production, but plastics can also adsorb POPs during their usage. In addition, recycling activities and incineration/combustion of plastics contribute to POPs environmental release (98). Several studies have investigated the interplay between POPs, GM dysbiosis and related disorders. For instance, polychlorinated biphenyls and organochlorine pesticides have proved to dysregulate the salt hydrolase activity of Bacteroidetes, Lactobacilli and Clostridia, thus influencing the bile acid metabolism. Furthermore, dichlorodiphenyldichloroethylene (DDE), a metabolite of the insecticide dichlorodiphenyltrichloroethane (DDT) with endocrine-disrupting and carcinogenic potential (99), increases Proteobacteria and Firmicutes/Bacteriodetes ratio, and enhances their synthesis of short-chain fatty acids, leading to pre-diabetes and obesity (100).

The exposure to air pollutants disrupts microbiota composition equilibrium, favouring dysbiosis and subsequently a “leaky gut” (101, 102). A recent systematic review by Van Pee et al. (103) has highlighted an inverse association between particulate air pollution exposure and the GM composition in human studies. In particular, air pollutants decrease the microbial diversity, increasing taxa belonging to Proteobacteria, Deferribacterota and Bacteroidetes, and reducing taxa belonging to Verrucomicrobiota. No univocal data emerge about their effect on Firmicutes and Actinobacteria in humans, as well as about their impact on microbial diversity indices and taxa in animals (103).

*Mucispirillum* is positively associated with PM_2.5_ exposure in older adults (103). *Mucispirillum* degrades mucins, a constituent of the intestinal mucus layer, essential for commensal flora homeostasis (104).

Air pollutants, on one hand, can negatively influence *Akkermansia muciniphila*, a bacterium with anti-inflammatory properties, able to reinforce the gut barrier (105, 106); on the other hand, can increase relative abundance of Campylobacter, whose species are often pathogenic for the gastrointestinal tract (e.g., *Campylobacter jejuni*) (107). Air pollutants-mediated gut damage and inflammation consist in focal epithelial desquamation, lower mucin 2 (Muc2) levels and an increase in colonic granulocyte infiltration (108, 109). Son et al. (110) demonstrated that PM can interfere with protein metabolism and calcium signalling through intestinal epithelium inflammation.

Li et al. (111) conducted a multiomics analysis in order to clarify the molecular mechanisms through which air pollutants alter the GM and metabolism in COPD patients. They found that PM_2.5_ decrease the microbial α-diversity in concentration-dependent manner. Moreover, some of the serum and fecal metabolites linked to air pollution (e.g., 2,5-furandicarboxylic acid) are also associated to disorders in lipid and fatty acid metabolism in COPD patients (111).

Gut microbiota is modified also by water and land pollutants. Guo et al. (20) found that antibiotics, especially tetracyclines, are widely present in the waterbodies of a typical water-rich Chinese city, with subsequent concerns about the safety of drinking water (112). Among the water pollutants, heavy metals, such as arsenic and cadmium, disrupt the microbiome and the metabolome with lack of diversity in microbiota composition. After cadmium and arsenic exposure respectively, Li et al. (86) identified worthy changes in 5 and 2 phyla, and 42 and 24 genera, such as the upregulation of *Barnesiella, Alistipes, Alkalitalea* and *Prevotella*. Furthermore, several butyrate-producers significantly decreased and a reduction of metabolite interactions was observed (86). These changes may contribute to the onset of type 2 diabetes and other metabolic disorders (113). Metal pollution, like lead, cobalt and zinc, also has effects on the selection of some antibiotic-resistant microbial communities (87, 114). Lead increases the abundance of Marvinbryantia and Ruminococcus and reduces the α-Proteobacteria. In addition, mercury, a potential contaminant of drinking water and fish, since it can accumulate in the wastewater of factories and agricultural activities, can change the GM composition in exposed mice. In fact, a reduced amount of Proteobacteria and Bacteroidetes, and a higher abundance of *Clostridium, Treponema, Lactobacillus* and *Helicobacter* have been described after mercury exposure (115). The reduction of *Bifidobacterium*, a beneficial microbe that preserves human gut barrier and local immune regulation (116), causes cadmium-dependent intestinal inflammatory responses. In fact, exposure to cadmium-containing pollutants has been observed to impair intestinal barrier and promote inflammation with decreased amount of *Bifidobacterium*, *Lactobacillus*, and fecal SCFAs (117).

Diesel exhaust particles inhalation also induces colonic epithelium inflammatory injury in mice, with a reduction of Lactobacilli. Notably, mice transplanted with DEP-treated donors’ microbiota developed the same colonic damage, and Lactobacilli resulted beneficial when administered as probiotic supplements (118). Seidenath et al. (119), exposing the bumblebee *Bombus terrestris* to oral DEPs, detected significant alterations in GM composition (e.g., higher abundance of the core bacterium *Snodgrassella*) and changes in gene expression related to signal transduction and metabolism, aimed at stress response. Liu et al. (120) have recently correlated the structural changes in murine GM after DEP exposure (e.g., the relative abundance of Bacteroidetes and Firmicutes, and their ratio) to obesity. They also found increased concentration of intestinal SCFAs, equally related to weight gain (120).

In literature, it has been observed that PFAS affect the intestinal microbiota. BPA is commonly used in the production of food containers, and other plastic products (121, 122), and it has been measured in several body fluids (123, 124). In animals, BPA leads to an alteration of Bacteroidetes/Firmicutes equilibrium. Furthermore, it is considered an endocrine disruptor, able to alter the reproductive function in both sexes, and cause cortisol imbalance (125).

Ma et al. (84) have recently studied the effects on gut barrier of bisphenol P (BPP), when present in foods. They exposed mice to different concentrations of BPP, observing the development of microbial dysbiosis characterized by increased amount of Firmicutes, reduced Bacteroidetes, increased number of Proteobacteria (including pathogenic bacteria, such as *Salmonella, Campylobacter*, and *Helicobacter*), and reduced α-diversity. This condition led to an intestinal barrier disruption, through lipopolysaccharide (LPS)/TLR4/NF-κB pathway activation (84).

In a mother-infant study, Lamichhane et al. (126) evaluated the effect on GM of PFAS exposure before and after birth. Analyzing both maternal and infant samples, they demonstrated that the exposure to PFAS correlates with a higher abundance of *Methanobrevibacter smithii* in maternal stool. The authors noticed a weaker influence of these chemicals on the infant microbiota (126). However, an association between prenatal PFAS exposure, particularly perfluorohexane sulfonic acid (PFHxS), gut microbiota changes and neurobehavioral disorders has been described in children. PFHxS levels correlate with a higher abundance of *Enterococcus* spp. and lower SCFA-producing species. Moreover, PFHxS-induced changes in α-diversity positively correlate with conduct problems (127).

## 4 Pollution, inflammatory bowel diseases and colorectal cancer

### 4.1 Inflammatory bowel diseases

In the last decades, inflammatory bowel diseases have been extensively studied. Both genetic and environmental factors contribute to their pathogenesis (128), as an example of the interplay between environmental exposure and genetic predisposition in human health and disease (129, 130).

Among the environmental factors, pollutants, such as particulate matter, NO₂, SO₂ and toxicants in drinking water (e.g., heavy metals or chemicals) have been associated to gastrointestinal (GI) damages leading to IBD (131–136). In addition, ionizing radiation can represent a probable trigger of gastrointestinal inflammation due to the consumption of involved food and water (137, 138).

The association between air pollutants, such as sulfur or nitrogen dioxide, and IBD incidence has been established in infants, while the causative effect of PM and O_3_ is still uncertain (139–141).

Intestinal mucosa can be exposed not only to dietary food and water pollutants, but also to air pollutants reaching the GI tract via mucociliary clearance from lungs (142, 143). In the gut, inhaled pollutants lead to lipid oxidation, microbiota disruption, impairment of epithelial barrier integrity and production of bacterial metabolites with systemic metabolic consequences (101). Overall, particles-induced gut inflammation is the result of both direct effects on intestinal epithelial cells and the release of toxic metabolites by gut microbes. The following production of ROS damages the epithelial tight junctions, increasing the gut permeability. For this reason, PM and microbial products enter the lamina propria and interact with the immune cells at this level, triggering a pro-inflammatory response that further increases the intestinal permeability (144). Dysbiosis can be responsible for intestinal inflammation (145); indeed, patients affected by IBD have a depletion of commensal microbes, not observed in healthy people (146).

Long-term exposure to coarse particles (PM_10_) determined epithelial damage and mucosal granulocyte infiltration, in IL-10^−/−^ mice, a commonly used IBD model. These modifications are accompanied by changes in their gut microbiome with an overgrowth of Firmicutes and Verrucomicrobia, and a decrease of Bacteroidetes and SCFA-producing bacteria. The butyrate depletion causes a loss of its intestinal and systemic beneficial effects, inducing gut barrier dysfunction and pro-inflammatory responses (52, 147).

The ingestion of ultrafine particles (diameter < 100 nm) increases Verrucomicrobia, decreases Actinobacteria, Cyanobacteria, and Firmicutes, with subsequent intestinal inflammation due to an increased intestinal infiltration by macrophages and neutrophils in low density lipoprotein receptor (LDLr)^−/−^ mice (148).

It has been observed that the inhalation of diesel exhaust particles promotes the release of Th1-related cytokines, such as TNF, in healthy volunteers, as well as oxidative stress at both systemic and colonic level in animal studies (149, 150). Interestingly, the exposure to DEPs has proved to increase the production of inflammatory cytokines and promote the ROS/extracellular signal-regulated kinase (ERK)/cFos pathway in human mesenchymal stem cells. Moreover, DEP-dependent ROS injury reduces the therapeutic potential of these cells in colitis mice (151).

Several studies have evaluated the relationship between long-term exposure to air pollution and gut inflammation. Conversely, Noviello et al. (152) investigated the effects of short-term exposure to air pollutants, especially PM_10_, on the incidence of IBD flares. Interestingly, the authors observed a positive correlation between the hospitalization for IBD in men with left-sided ulcerative colitis and the levels of air pollution to which they were exposed in the previous days (152).

Benzo[a]pyrene, a toxicant belonging to the family of polycyclic aromatic hydrocarbons, induces intestinal inflammation through a disequilibrium in bacterial populations towards an inflammatory subset. In fecal samples of mice exposed to BaP, *Lactobacillus,* which presents anti-inflammatory functions (153, 154), and Ruminococcaceae, producing SCFAs (155), were both reduced. Furthermore, bacteria belonging to the family of Alcaligenaceae, correlated to ulcerative colitis (156), increased after BaP exposure (157). On the other side, Adachi et al. (158) evaluated the effects of oral BaP on a mouse IBD model. They found that BaP reduces the weight loss, the disease activity, the histological injury, the pro-inflammatory gene expression and the plasma levels of IL-6 in colitis mice (158). A cross-sectional analysis of a large adult sample in the United States found a significant correlation between PAH exposure and bowel disorders (159).

At gut level, heavy metals, such as arsenic, cadmium, chromium, lead and mercury, have been associated to oxidative stress, gut microbial changes and inflammation, leading to intestinal barrier dysfunction (160). In a study analyzing deciduous teeth, prenatal lead exposure has been associated with future risk of IBD (161). Several studies evaluated the heavy metal concentration in samples of ulcerative colitis patients, showing high levels of lead and copper in serum, and high levels of zinc and iron in hair and gut, respectively. In addition, the iron exposure could exacerbate the symptoms of the disease (162).

Yin et al. (163), using a colitis mouse model, found that BPA and fluorene-9-bisphenol treatments significantly altered sugar and fatty acid metabolisms, respectively. Additionally, they both induced inflammatory responses, suggesting a role of these environmental pollutants in ulcerative colitis development (163). It has been observed that a chronic exposure to BPA, rather than a single intake of contaminated food, negatively impact on gut health. In IBD patients, BPA serum concentrations increase during the active periods of disease and particularly in colonic forms (115). An observational study on patients with Crohn’s disease confirms these data, showing that G protein-coupled estrogen receptor (GPER) expression increases in patients with active disease and correlates with BPA levels. Additionally, BPA levels correlate to serum IL-23 and endotoxin levels, thus revealing an important role of this endocrine disruptor on intestinal barrier impairment and systemic inflammation in IBD (164). Malaisé et al. (165) exposed mice to BPA, bisphenol S (BPS) or bisphenol F (BPF) in order to outline their effects on food tolerance and the potential risk of IBD. Their results showed that BPs impair oral tolerance to ovalbumin through increased humoral and inflammatory cell-mediated responses. Only BPF was associated to colitis exacerbation in mice, higher TNF-α level in colon and reduced fecal immunoglobulins A (IgA) (165).

**Figure 1:**
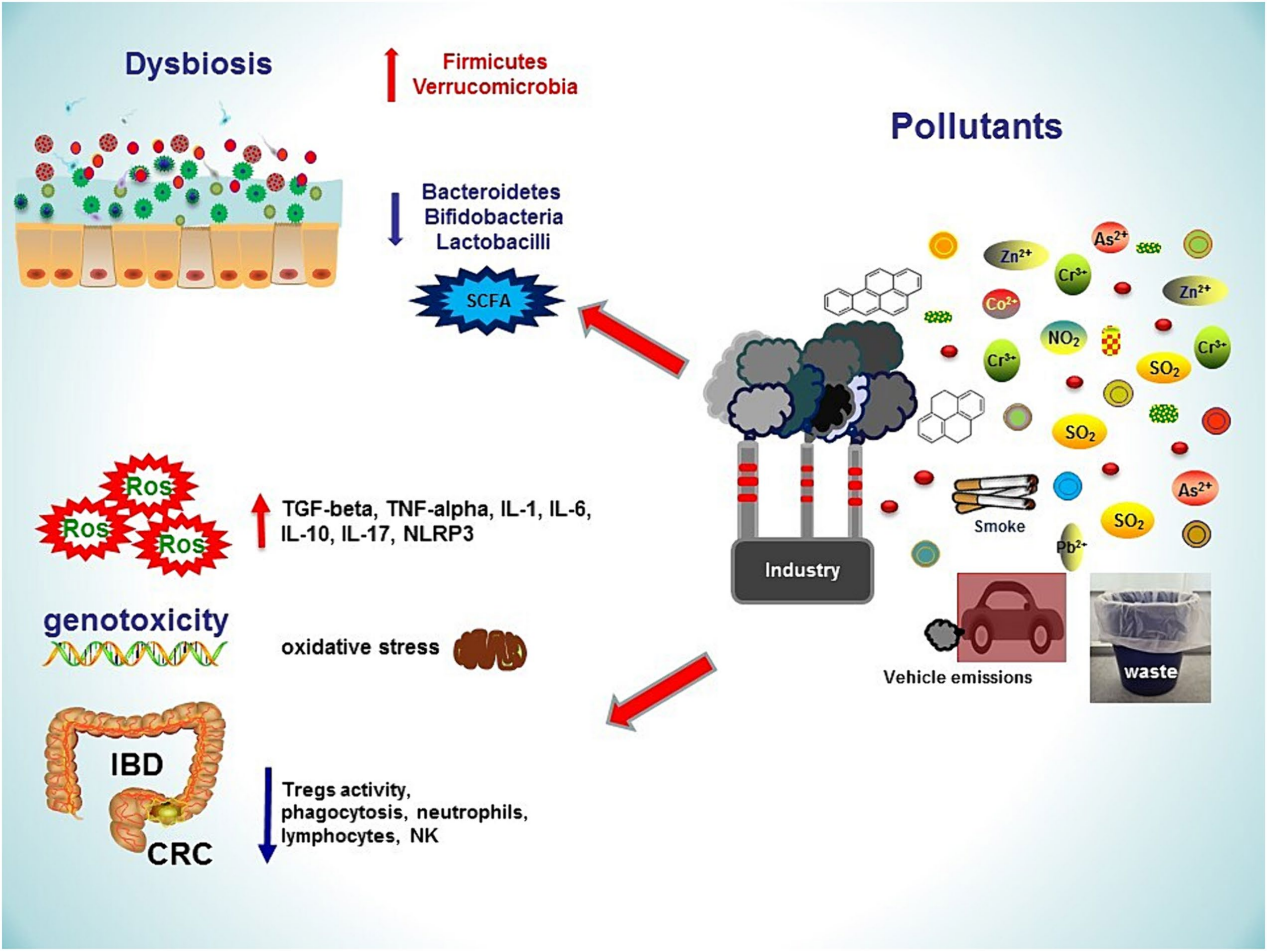
Several sources of environmental pollution lead to gastrointestinal damages, inflammatory bowel diseases and colorectal cancer. Pollutants induce dysbiosis by increasing Firmicutes and Verrucomicrobia, decreasing Bacteroidetes and butyrate-producing gut microbes. An impaired intestinal barrier function, together with oxidative stress and increased inflammatory responses, contribute to the development of gastrointestinal diseases. NO₂, nitrogen dioxide; ROS, reactive oxygen species; SO₂, sulfur dioxide; TNF, tumour necrosis factor; SCFA, short chain fatty acid; NLRP3, pyrin domain-containing protein 3; IL, interleukin; IBD, Inflammatory Bowel Disease; CRC, Colo-Rectal Cancer; Pb, Lead; As, Arsenic; Co, Cobalt; Zn, Zinc; Cr, Chromium.

Among the water pollutants, microplastics can be found ubiquitously in water sources and the prolonged exposure to them through diet induces intestinal barrier impairment, microbial disequilibrium, and metabolic alterations, thus triggering gut inflammation. Furthermore, individuals with pre-existing gastrointestinal disorders present more serious consequences, compared to those without them (79). MPs exert immunotoxic effects by inducing oxidative stress, and impairing immune cell chemotaxis and humoral responses, thus weakening the antimicrobial ability of the immune system (166). In a mouse model, Xie et al. (167) noticed that MP exposure induces an over proliferation of colonic mucosa, increasing the amount of intestinal stem cells and the expression of proliferating cell nuclear antigen (PCNA) and c-Myc oncogene. Moreover, MPs worsen the clinical presentation and the histological damage of colitis (167). Interestingly, Yan et al. (168) found that fecal MP concentrations (especially polyethylene terephthalate and polyamide) in patients affected by IBD were higher than in the healthy group and correlated to disease severity. Plastic containers and dust have been identified as the most important sources of MPs (168). A recent prospective cohort study including more than 22 thousand participants free from IBD, conducted by Zhou et al. (169), demonstrated that drinking water contaminated with metals (e.g., mercury, manganese), disinfectants or fertilizers increased the IBD risk (Figure 1).

### 4.2 Colorectal cancer

Colorectal cancer is the second cause of death for cancer in the world (170). It has been well established that chronic inflammation can lead to carcinogenesis; therefore, ulcerative colitis and Crohn’s disease are risk factors for CRC (171, 172), as example of inflammation-related tumorigenesis. Chronic intestinal inflammation can induce oxidative stress and DNA damage. The result is the inflammation-dysplasia-carcinoma sequence (173, 174). In this context, gut microbiota seems to play a key role, due to development of dysbiosis and subsequent chronic inflammation. In particular three bacterial species, *Fusobaterium nucleatum*, *Escherichia coli* and *Bacteroides fragilis*, seem to be correlated with the process of colorectal carcinogenesis in humans (175). Bacterial communities, such as mucus-invasive biofilms, rather than individual organisms, are relevant in intestinal carcinogenesis, particularly when they interact with host and environmental factors (176).

Other risk factors for CRC are represented by diet, obesity, genetic factors (177) and environmental pollutants. A recent study has shown that the residents near industries releasing air pollutants (e.g., nonylphenol, naphthalene, antimony, manganese, organotin compounds, dichloromethane, and vanadium) had higher risks to develop CRC than the ones near industries responsible for water pollution (178).

In a comprehensive meta-analysis, Fu et al. (78) detected a strong association between the exposure to fine particulate matter (PM_2.5_) and an increased risk of CRC. They reported higher incidence and mortality in North America, particularly in the United States, compared to Asia (78). In addition, a study aimed at evaluating the effect of 10-year exposures to air pollutants on primary cancer incidence in American adults, revealed an association between PM_2.5_ and NO_2_ exposure and the development of CRC (179). At the same time, the use of pesticides and herbicides (e.g., trifluralin) has been reported to be associated to CRC (82, 180, 181). Xie et al. (182) found the existence of correlations between lifetime days of herbicide and insecticide exposure and the risk of CRC. Abolhassani et al. (183) showed that organochlorine pesticides can promote the methylation of the promoter of the onco-suppressor gene P16, thus reducing its expression and eventually lead to the development of CRC. In a case–control study, Lee et al. (184) described the carcinogenetic potential of chronic exposure to low-dose POPs. Evaluating the serum concentrations of organochlorine pesticides and polychlorinated biphenyls, as examples of POPs, the authors found their association with colorectal polyps and CRC. As highly lipophilic chemical mixtures, POPs can accumulate in adipose tissue and be released into circulation (184).

Since various epidemiological studies have recognized the association between the exposure to PFAS and cancer, Cui et al. (185) examined the potential link between PFAS exposure and CRC prognosis. In a study enrolling more than 300 patients at Beijing Hospital in China, the authors detected a positive correlation between PFAS serum concentration and the number of metastatic lymph nodes. These data suggest a negative prognostic impact of PFAS on CRC patients (185).

The chlorination of drinking water has been studied for its possible association with the development of CRC and bladder cancer (186, 187). During the process of chlorination, halogenated chemicals and hydroxyfuranone obtained from chlorine reactions have showed carcinogenetic activity (188, 189). A matched case–control study in Ethiopia has demonstrated that drinking chlorinated water for years is a risk factor for CRC (190). Helte et al. (191) evaluated the incidence of CRC in a large population with a long-term exposure to trihalomethanes (THMs), disinfection by-products in chlorinated drinking water. They observed a higher risk of CRC in exposed men compared to non-exposed, particularly for proximal colon cancer. No association between THMs and CRC was found in women (191).

Bonfiglio et al. (88) shed light on the relationship between toxic metal bioaccumulation in human body and the risk of CRC. They described several mechanisms of toxicity, often involving oxidative stress, enzyme inhibition, dysregulation of cellular signalling pathways and DNA damage. For instance, lead increases the intracellular production of ROS that exceed the physiological antioxidant capacity, thus causing damage to proteins, lipids and DNA. A chronic exposure to low doses of aluminum results in oxidative stress in colon cells, leading to chronic inflammation and genomic instability. Chromium exposure impairs DNA repair mechanisms and decreases the expression of p53, thus disrupting cell cycle control and promoting cell proliferation. Similarly, cadmium stimulates cell proliferation and survival responses at low doses, whereas it induces cell death at higher doses (88). At the same time, a systematic review by Katsas et al. (192) confirms the association of chromium exposure with gastric and CRC.

The BPA is associated to CRC development, promoting epithelial to mesenchymal transitions (193). BPA exposure also leads to the upregulation of fascin (193), which can be involved in the chemotherapeutic resistance (194–196). Hong et al. (197) reported, through a lipidomic approach, that tumour tissue lipid metabolites (e.g., glycerophospholipid metabolism pathway) may play an important role in the link between BPA and CRC. Deng et al. (198) suggested that this association could be explained by the oxidative stress caused by BPA and its interactions with genetic variants. As observed by Xia et al. (199), BPA exposure promotes a disproportionate production of ROS through NADPH oxidase (NOX) and mitochondrial electron-transport chain (ETC), activating in turn the HIF-1α/VEGF/PI3K/AKT (hypoxia-inducible factor-1alpha/vascular endothelial growth factor/phosphoinositide 3-kinases/Ak strain transforming) axis and inducing the invasiveness of colon cancer cells. BPA exposure has been linked with increased mortality in adults. However, urinary BPA levels seem to be U-shaped associated with cancer mortality. Interestingly, Yuan et al. (200) found that, at the same time, extremely low levels of BPA could negatively impact on cancer survival, probably due to a role of BPA in regulating cell cycle and apoptosis in tumour cells.

In scientific literature, contrasting data about acrylamide, a possible carcinogenic compound to humans according to the International Cancer Research Center, can be found. Acrylamide is a contaminant formed by thermal process of various foods (e.g., coffee, French fries, bread and biscuits). Several epidemiological studies have been conducted, most of them showing no clear association with the risk of CRC (201).

Dioxin compounds (202) show carcinogenetic effects through the aryl hydrocarbon receptor, a transcription factor, also known as dioxin-receptor (203). The AhR can metabolize PAHs to highly reactive carcinogenic intermediates. Moreover, acting as a sensor also for GM metabolites, the AhR plays a role in microbe-mediated tumorigenesis through several mechanisms (e.g., the Wnt/β-catenin signalling pathway), promotes immune tolerance in tumour microenvironment, and tumour metastasis (204).

Since the GI tract is an entry point for micro-and nanoplastics (MNPs), modern research has been progressively clarifying their relationship with CRC. Cetin et al. (80) found a higher number of MPs, such as polyethylene and polyamide, in tumoral colon tissues, compared to non-tumoral colon tissues. Indeed, various studies have focused on the carcinogenic potential of MNPs, able to promote inflammation and genotoxicity, as well as accumulate in cells and tissues (81). Brynzak-Schreiber et al. (205) evaluated the effect of these pollutants on different CRC cell lines. The uptake of polystyrene micro-and nanoplastics (PS-MNPs) and their distribution to daughter cells were observed. Interestingly, the smallest particles promoted cell migration, thus suggesting a role of MNPs in tumour progression (205). Li et al. (206) noticed that the increased environmental exposure to MPs is temporally correlated with a higher number of early onset (under 50 years of age) CRC. The authors suggest that MPs reduce the protective effect of the intestinal mucus layer, thus increasing the likelihood of carcinogenesis (206).

## 5 Future directions

As discussed above, environmental pollution takes part in an intense crosstalk with the host immune system and the gut microbiota, which could explain the development of chronic diseases.

Further studies with advanced molecular biologic techniques could provide novel insights into the potentially serious effects on health by pollutants and clarify the molecular pathways involved in the onset and the exacerbation of these diseases. Researchers should evaluate the underlying biological mechanisms (e.g., immune modulation, genetic and epigenetic changes) as a complex interplay, rather than independent mechanisms. The exposome, which encompasses environmental factors and individual behaviours responsible for biological processes, should be integrated into multi-omics analysis approaches. Indeed, the environment is closely linked to genome, epigenome, transcriptome, proteome, and metabolome (130).

In this context, a fundamental prerequisite is an enriched awareness about the GM composition and functions. As suggested by Shalon et al. (207), ingestible devices collecting samples from different intestinal regions could better clarify the longitudinal and temporal variability of GM and the physiopathology of the GI tract, compared to the stool analysis alone.

Whether interventions on gut microbiota (e.g., the use of probiotics, dietary fiber consumption, SCFAs supplements, etc.) could prevent or reduce the impact of inflammation in pollution-induced IBD and CRC would be of future interest for investigation. The term probiotics refers to non-pathogenic microbes, like lactic acid bacteria, which provide benefits on human health when appropriately administered (208). On the other side, prebiotics are insoluble fibers that induce the intestinal overgrowth of positive bacteria, contribute to nutrient absorption and play a role in immune modulation (209).

For instance, *Lactobacillus* can be used as anti-inflammatory drug in IBD patients (210), showing ability of inhibiting carbonic anhydrases, responsible for intestinal damage (118), and NF-κB pathway (211). Lactobacilli can secrete lactoceptin, which selectively degrades pro-inflammatory chemokines (212), and reduce expression of TNF-α-converting enzyme with a consequent suppression of macrophage inflammatory mediators (213).

Simon et al. (214) suggested a beneficial role of probiotics, such as *Bacillus megaterium,* acting as a chelant of heavy metals, mercury and lead, and degrading, among others, ammonia and nitrophenol. Moreover, *Bacillus megaterium* demonstrated antioxidant and antimicrobial activity against pathogens (214). Zhang et al. (215) explored the reproductive toxicity of MPs in mice and the potential therapeutic effect of probiotics. They found that mice exposed to MPs had testicular dysfunction and changes in GM composition (lower abundance of *Lactobacillus* and higher abundance of *Prevotella*). Probiotics supplementation downregulated the IL-17 signalling pathway and the inflammatory response improving reproductive function (215).

Furthermore, in diseases caused by PM exposure, dietary fibers or SCFAs supplements could have beneficial effects as a therapeutic tool (216). SCFAs, deriving from dietary fiber fermentation by GM, are essential to maintain intestinal homeostasis and modulate systemic immune responses. Cheng et al. (216) suggested to target the SCFA/G-protein-coupled receptor (GPCR) signalling through SCFAs supplements, in order to reduce the air pollution–related inflammation and prevent the lung-gut axis disruption. Román-Ochoa et al. (217) reported that dietary fibers (e.g., wheat bran and pectin) reduce the negative impact of heavy metals, such as arsenic, cadmium and mercury, on human gut microbiota *in vitro*, thus suggesting their potential role in reducing metal-induced GM dysbiosis *in vivo*.

Undoubtedly, in future it is essential to shape public health policies to decrease the levels of environmental pollutants and their effects on microbiota with subsequent activation of pro-inflammatory pathways. In this context, several strategies have been considered, including the increase of green spaces in urban areas, the promotion of public transportation rather than car driving, the reduction of industrial emissions and a careful activity of the measuring stations (20). Yang et al. (218) studied the consequences of environmental regulation in China. They found that the Two Control Zones (TCZ) policy have positively influenced public health, by reducing environmental pollution levels and the incidence of respiratory diseases in the TCZ areas (218).

Additionally, it is essential to raise awareness in general population about the negative effects of pollutants on human health, such as cancer, through appropriate government campaigns (63).

## 6 Conclusion

Pollution is a relevant concern of modern society for its effects on human health. Several studies report that environmental pollution is implicated in the pathogenesis of chronic inflammatory disorders and cancer, describing the potential molecular mechanisms involved in the health-disease transition.

Pollutants can affect the activity of immune cells, perturbing the immune regulation and triggering pro-inflammatory responses. The exposure to several pollutants also leads to alterations in microbiome, such as a decreasing abundance of beneficial microbes and an overgrowth of pro-inflammatory species, with a subsequent increased intestinal permeability contributing to the onset of intestinal disorders.

Pollutants, immune system and gut microbiota represent the perfect trio able to induce and promote severe diseases such as IBD and CRC.

## Author contributions

PR: Conceptualization, Methodology, Writing – original draft, Writing – review & editing. AG: Supervision, Validation, Writing – review & editing. GG: Supervision, Validation, Writing – review & editing. RC: Conceptualization, Methodology, Writing – original draft, Writing – review & editing.
